# Strategies for Prevention of Varicella Pneumonia: A Cost-Effectiveness Analysis

**DOI:** 10.1155/S1064744996000166

**Published:** 1996

**Authors:** George A. Macones, Stephanie Ewing, Neil S. Silverman

**Affiliations:** 1 Center for Clinical Epidemiology and Biostatistics University of Pennsylvania School of Medicine Room 901, Blockley Hall 423 Guardian Drive Philadelphia PA 19104-6021 USA; 2 Department of Obstetrics and Gynecology Thomas Jefferson University Hospital Philadelphia PA USA

## Abstract

*Objective:* The objective of this study was to compare the cost-effectiveness of 3 strategies of serologic enzyme-linked immunosorbent assay (ELISA) testing and post-exposure varicella zoster immune globulin (VZIG) prophylaxis for the prevention of maternal varicella pneumonia during pregnancy in patients with *negative* or *uncertain* histories of varicella infection.

*Methods:* A decision tree was constructed to compare the following strategies: 1) routine serologic testing for varicella immunity followed by targeted post-exposure VZIG prophylaxis, 2) post-exposure serologic testing followed by targeted VZIG prophylaxis, and 3) untargeted post-exposure VZIG administration. The probabilities for the model were obtained from the medical literature and supplemented by expert opinion. The costs were obtained by a review of inpatient hospitalizations for varicella pneumonia. All costs were converted to 1995 dollars.

*Results:* Routine serologic testing followed by targeted post-exposure VZIG prophylaxis was the most costly strategy ($37.22/person), with no demonstrable increase in benefit compared with the other 2 strategies. The disutility of this strategy compared with the others was stable across a wide range of values for the probabilities and costs utilized in the sensitivity analysis. We were unable to differentiate between the cost-effectiveness of the other 2 strategies.

*Conclusions:* Routine serologic testing for varicella immunity in patients with negative or uncertain histories of varicella infection should not be performed. The remaining options of screening and prophylaxis appear to be reasonable alternatives for dealing with varicella exposures.


Infectious Diseases in Obstetrics and Gynecology 4:71–76 (1996)
© 1996 Wiley-Liss, Inc.

### Strategies for Prevention of Varicella Pneumonia: A Cost-Effectiveness Analysis

**George A. Macones, Stephanie Ewing, and Neil S. Silverman**

*Center for Clinical Epidemiology and Biostatistics, University of Pennsylvania School of Medicine, and Department of Obstetrics and Gynecology, Thomas Jefferson University Hospital, Philadelphia, PA*

### Abstract

*Objective:* The objective of this study was to compare the cost-effectiveness of 3 strategies of serologic enzyme-linked immunosorbent assay (ELISA) testing and post-exposure varicella zoster immune globulin (VZIG) prophylaxis for the prevention of maternal varicella pneumonia during pregnancy in patients with *negative* or *uncertain* histories of varicella infection.

*Methods:* A decision tree was constructed to compare the following strategies: 1) routine serologic testing for varicella immunity followed by targeted post-exposure VZIG prophylaxis, 2) post-exposure serologic testing followed by targeted VZIG prophylaxis, and 3) untargeted post-exposure VZIG administration. The probabilities for the model were obtained from the medical literature and supplemented by expert opinion. The costs were obtained by a review of inpatient hospitalizations for varicella pneumonia. All costs were converted to 1995 dollars.

*Results:* Routine serologic testing followed by targeted post-exposure VZIG prophylaxis was the most costly strategy ($37.22/person), with no demonstrable increase in benefit compared with the other 2 strategies. The disutility of this strategy compared with the others was stable across a wide range of values for the probabilities and costs utilized in the sensitivity analysis. We were unable to differentiate between the cost-effectiveness of the other 2 strategies.

*Conclusions:* Routine serologic testing for varicella immunity in patients with negative or uncertain histories of varicella infection should not be performed. The remaining options of screening and prophylaxis appear to be reasonable alternatives for dealing with varicella exposures. © 1996 Wiley-Liss, Inc.

Key words
Maternal varicella, chickenpox, ELISA screening, VZIG

Varicella (chickenpox) is a highly contagious viral illness usually acquired in childhood. Previous studies have shown that 90–95% of adults born in North America are serologically immune.[1] While the disease is mild in childhood, it can cause significant morbidity in adults. In addition, these infections are believed to be especially severe during pregnancy because of alterations in the maternal immune status. Varicella infection in pregnancy has been associated with preterm labor, congenital malformations, and maternal pneumonia.[2,3] Pneumonia is the most common complication in adults, with a mortality rate approaching 25%. While the actual number of cases of maternal varicella pneumonia is small, the severity of this disease warrants interventions and strategies for prevention.

The administration of varicella zoster immune globulin (VZIG) within 96 h of exposure has been shown to prevent or ameliorate the effects of varicella infection in susceptible individuals.[4] Although VZIG does not appear to prevent congenital infection, many experts have recommended the administration of VZIG to all susceptible pregnant women exposed to varicella-infected individuals in order to prevent the complications associated with overwhelming maternal infection. There are several dif-

**Address correspondence/reprint requests to Dr. George A. Macones, Center for Clinical Epidemiology and Biostatistics, University of Pennsylvania School of Medicine, Room 901, Blockley Hall, 423 Guardian Drive, Philadelphia, PA 19104-6021.**

Received January 19, 1996
Accepted May 29, 1996

ficulties in practice with this recommendation. First, the susceptibility must be ascertained accurately within a short time if VZIG is to be given within 96 h after exposure. Such rapid testing is not available in most areas. Second, VZIG is difficult to obtain and is perceived as "expensive." As such, physicians are unlikely to prescribe this medication unless a lack of immunity is relatively certain. Because of these problems, several authors have evaluated the patient history as a predictor of true serologic immunity.[5,6] It has been demonstrated that a positive maternal history of varicella infection reliably predicts true serologic immunity.[5,6] However, in patients with negative or uncertain histories, the relationship to true immune status is less reliable. Thus, there are several obvious options for dealing with varicella exposure in patients with negative or uncertain histories of varicella infection:

1. Routine serologic [enzyme-linked immunosorbent assay (ELISA)] screening at the initial prenatal visit, followed by targeted post-exposure VZIG administration.
2. Post-exposure serologic testing, followed by targeted VZIG administration.
3. Untargeted post-exposure VZIG administration.

In cases of clinical and economic uncertainty, a cost-effectiveness analysis can be a useful tool in guiding clinical practice.[7,8] The purpose of this study was to apply this methodology to the comparison of the above strategies for the prevention of varicella pneumonia in patients with negative or uncertain histories of varicella infection.

### MATERIALS AND METHODS

A decision tree was created to compare the options of 1) routine varicella ELISA testing, 2) post-exposure testing with targeted VZIG administration, and 3) untargeted post-exposure VZIG administration in pregnant patients with negative or uncertain histories of varicella infection. This decision tree was created and analyzed using Data 2.5 software (TreeAge Software, Boston, MA).

### Strategies

A graphic representation of the 3 strategies is shown in Figure 1.

#### *Strategy 1*

In the strategy of routine varicella ELISA testing, we assumed that every patient with a negative or an uncertain history of infection would have a varicella-titer determination at the initial prenatal visit. In the case of a patient exposed to a varicella-infected person ("household" contact), we assumed that a patient with a positive titer would not receive VZIG, while a patient with a negative titer would receive VZIG.

#### *Strategy 2*

In the strategy of post-exposure testing with targeted VZIG administration, we assumed that a patient would not have a varicella ELISA unless she was exposed to a varicella-infected individual ("household" contact). In the latter case, we assumed that a patient with a positive titer would not receive VZIG, while a patient with a negative titer would receive VZIG. We also assumed that the results of the varicella ELISA would always be available quickly enough for the administration of VZIG within 96 h of exposure.

#### *Strategy 3*

In the strategy of untargeted post-exposure VZIG administration, we assumed that no patient would ever be tested for varicella immunity even if she was exposed to an infected individual. In this strategy, we assumed that all patients with negative or uncertain histories exposed to varicella-infected individuals would receive VZIG empirically. We also assumed that VZIG would always be given within 96 h of exposure.

### Probabilities

The probabilities and ranges for this decision tree were obtained from a review of the pertinent literature (Table 1).[3–6,9–22] While several of these probability estimates are based on data from several decades ago,[2,4,14–17] they represent the best estimates of these probabilities available. We also used wide ranges for these probabilities in our sensitivity analysis to account for this uncertainty. For the efficacy of VZIG, we sought expert opinions from subspecialists in infectious disease because of a lack of precise data on VZIG efficacy in pregnancy. To evaluate the role of uncertainty for this probability, we used a wide range for this probability estimate in our sensitivity analysis.

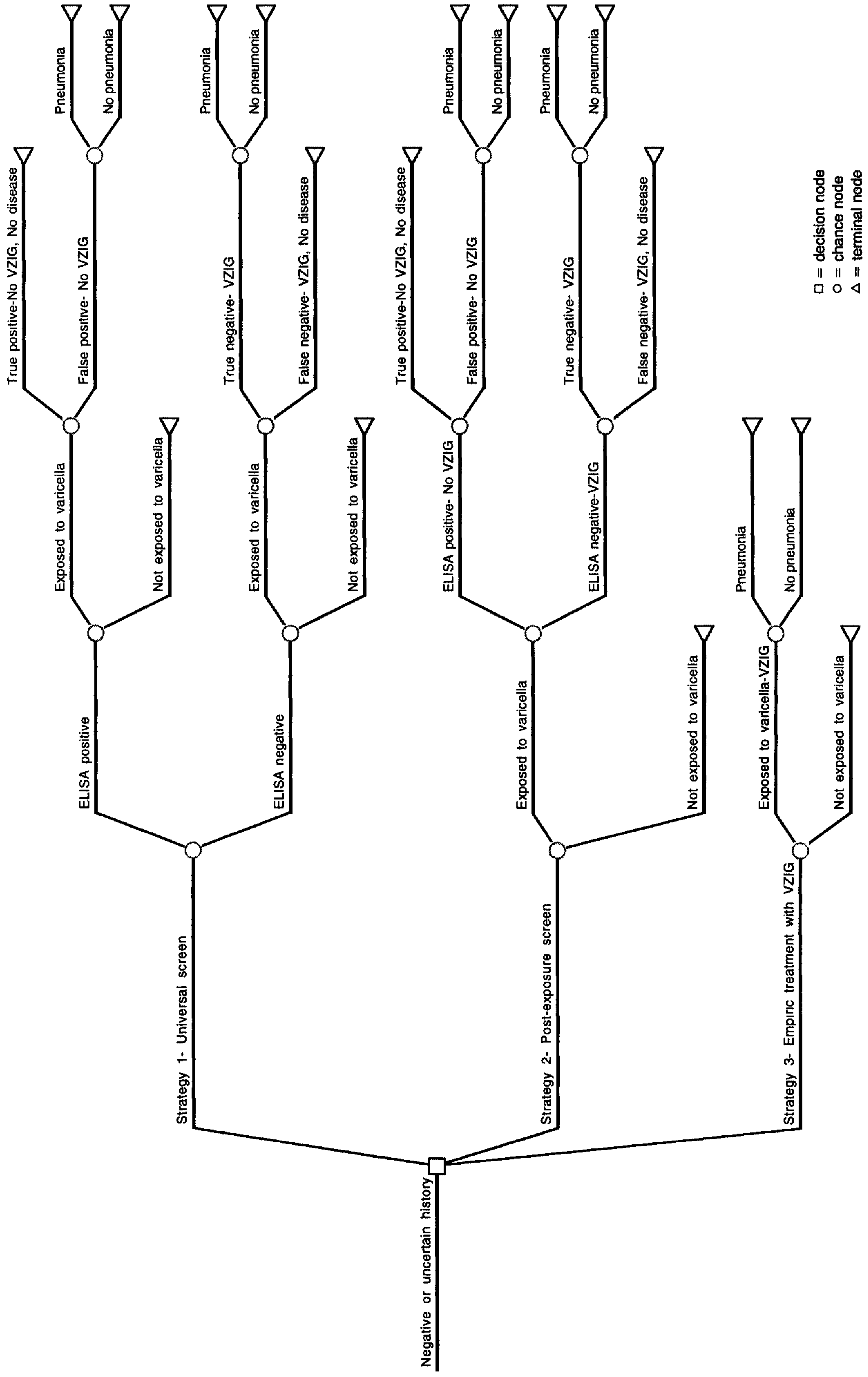

Fig. 1. The decision tree.

TABLE 1. Probabilities for the model

| | Base case | Range |
|---|---|---|
| Serologic immunity[5,6] | 0.65 | 0.55–0.80 |
| Varicella exposure[9,10] | 0.0005 | 0.0001–0.02 |
| ELISA sensitivity[11–13] | 0.87 | 0.70–0.97 |
| ELISA specificity[14,15] | 0.90 | 0.84–1.00 |
| Varicella after exposure ("household" contact)[14,15] | 0.80 | 0.70–0.95 |
| Varicella pneumonia/−VZIG[3,16–19] | 0.10 | 0.05–0.30 |
| Varicella pneumonia/+VZIG[4,20–22] | 0.05 | 0.01–0.20 |

### Costs

The cost of a varicella ELISA was based upon an average of the costs from several laboratories in the Philadelphia area. The cost of an average dose of VZIG was ascertained from the hospital pharmacy billing system. To obtain information about the cost of impatient hospitalization for varicella pneumonia, we conducted a medical-record search for the years 1984–1994 using ICD-9-CM codes for varicella pneumonia cross-linked to the codes for pregnancy. The charts identified with this search strategy were then briefly reviewed to ascertain the accuracy about diagnosis and pregnancy status. The charts of patients with underlying immune suppression were excluded. The inpatient charges for these patients were then obtained. Since these admissions were over a 10-year period, all dollars were converted to 1995 dollars by using a hospital-specific inflation factor. All charges were converted to cost by using a hospital-specific cost:charge ratio.

### Outcomes and Analysis

By folding back our decision tree for each of the 3 strategies, we calculated the average direct cost per patient and the percentage of cases of varicella pneumonia prevented for the specific strategy. Since we assumed the efficacy of VZIG to be 50%, no strategy could be perfectly effective in preventing varicella pneumonia. The best strategy would be the one with the lowest cost per patient and the highest percentage of cases of varicella pneumonia prevented. To test the impact of the plausible ranges of probabilities and costs on the clinical decision, we performed multiple one- and two-way sensitivity analyses.

### RESULTS

The baseline estimates, ranges, and references for the various probabilities used in the model are listed in Table 1. Our medical-record search revealed 8 patients with varicella pneumonia, none of whom were excluded from our cost calculation. The baseline cost (Table 2) for this disease was the average of the charges for the 8 patients. The range used in the sensitivity analysis included the lowest and highest charges for an individual patient.

TABLE 2. Costs used in the model

| | Base case | Range |
|---|---|---|
| Varicella ELISA | $37 | $21–77 |
| VZIG | $283 | $120–560 |
| Varicella pneumonia | $43,644 | $8,420–112,427 |

Overall, routine serologic testing (strategy 1) was the costliest option ($37.22/patient). Moreover, no additional benefit in the prevention of varicella pneumonia was associated with this strategy compared with the other strategies (Table 3). This result was stable across the range of plausible probabilities and costs utilized for the one- and two-way sensitivity analyses, meaning that routine serologic screening was never the preferred strategy under any circumstance. This result is clearly driven by the low exposure rate to varicella in pregnancy. However, even when the probability of exposure was varied over an extremely wide range, this disutility for strategy 1 persisted. Thus, routine screening should not be employed. This analysis of the impact of the exposure rate on the clinical decision also suggested that our results are generalizable to groups of individuals with higher exposure rates, e.g., women with occupational exposures or children in the home.

Post-exposure testing with targeted VZIG (strategy 2) and untargeted post-exposure VZIG administration (strategy 3) were very similar with regard to the average cost per patient and expected benefit, although strategy 3 would prevent approximately

TABLE 3. Cost per patient and effectiveness of strategies

| | Strategy 1 | Strategy 2 | Strategy 3 |
|---|---|---|---|
| Serologic testing | All | Post-exposure | None |
| Post-exposure VZIG | Selective | Selective | All |
| Cost per patient | $37.22 | $0.19 | $0.24 |
| % of varicella pneumonia prevented | 47 | 47 | 50 |

3% more cases of pneumonia. This difference in effectiveness is related to the imperfect test characteristics for varicella serologic testing. The differences between these 2 options were sensitive to many of the assumptions in our model, including the probability of exposure, probability of serologic immunity, and cost of disease. As we could not differentiate between the cost-effectiveness of these latter 2 strategies with our model, either strategy appears reasonable.

### DISCUSSION

The clinical scenario in which a potentially susceptible pregnant woman is exposed to varicella is of great concern to both the patient and her physician. For this reason, some investigators have suggested that a varicella ELISA screen be added to routine prenatal testing in women with negative or uncertain histories of varicella infection. Our analysis clearly demonstrated that routine screening was dominated by the other options in that it was the most costly strategy with no additional benefit compared with the other strategies. The model was robust in that "routine screening" was never a preferred strategy across the ranges used in any of the sensitivity analyses. For this reason, this strategy should not be employed.

The strategies of post-exposure serologic testing with selective treatment and empiric post-exposure prophylaxis were similar in both cost and clinical benefit, although empiric prophylaxis would prevent approximately 3% more cases of varicella pneumonia. This small difference in clinical benefit is related to the imperfect test characteristics of varicella ELISA testing. The small difference between these 2 strategies was sensitive to many of the assumptions examined in the sensitivity analysis. Thus, we are unable to make specific recommendations regarding these 2 options. Still, for the many areas where rapid testing is not available, empiric post-exposure treatment is the most cost-effective of the remaining 2 options.

Several assumptions of importance for interpretation were made in this model. First, there were no reports of controlled trials evaluating the efficacy of VZIG in pregnant women. We chose to use both the available evidence in nonpregnant patients supplemented by expert opinions from infectious disease specialists to estimate the efficacy of treatment. We also chose a wide range of values for use in the sensitivity analysis. Second, the cost was ascertained by a review of the inpatient admissions for varicella pneumonia, which possibly overestimates the true cost because not all cases of pneumonia will result in hospitalization. Again, even with a wide range of values chosen for the cost of disease, the principal conclusions of our study were unchanged in the sensitivity analysis. Third, we assumed that VZIG was not associated with any severe adverse effects. While there have been no reports of the transmission of infectious agents with VZIG, such an occurrence might dramatically change our results. Fourth, we assumed that VZIG did not prevent the congenital varicella syndrome, which is estimated to occur in 4% of first-trimester infections. This assumption is based on several case reports of this syndrome in infants of mothers treated with VZIG. However, there may be a clinical benefit to the fetus which has not yet been elucidated.

We did not consider the utility of the varicella vaccine in our analysis. While this vaccine is not recommended for use during pregnancy, a program of universal screening of adults (or of reproductive-age women alone) may be a cost-effective strategy. Questions about the long-term effectiveness of the vaccine in adults make this possibility speculative.

In summary, our study demonstrates that universal screening for varicella immunity followed by post-exposure treatment with VZIG in patients with negative or uncertain histories should not be performed. The choice between the other clinical strategies should be based on the availability of rapid serologic testing for varicella immunity.

### REFERENCES

1. Gershon AA, Rakin R, Steinberg S, et al.: Antibody to varicella-zoster virus in parturient women and their offspring during the first year of life. Pediatrics 58:692–696, 1976.
2. Harris RE, Rhoades ER: Varicella pneumonia complicating pregnancy: A report of a case and a review of the literature. Obstet Gynecol 25:734–740, 1965.
3. Paryani S, Arvin A: Intrauterine infection with varicella zoster virus after maternal varicella. N Engl J Med 314: 1542–1546, 1986.
4. Ross AH: Modification of chickenpox in family contacts by administration of gamma globulin. N Engl J Med 267:368–375, 1962.
5. McGregor JA, Mark S, Crawford GP, Levin MJ: Varicella zoster antibody testing in the care of pregnant women exposed to varicella. Am J Obstet Gynecol 157:281–284, 1987.
6. Silverman NS, Ewing SH, Todi N, Montgomery OC: Maternal varicella history as a predictor of varicella immune status. J Perinat 16:35–38, 1996.
7. Eisenberg JM: Clinical economics: A guide to the economic analysis of clinical practices. JAMA 262:2879–2886, 1989.
8. Drummond MF, Stoddart GL: Methods of economic evaluation of health programmes. World Health Stat Q 38:355–367, 1985.
9. Sever J, White LR: Intrauterine viral infections. Annu Rev Med 19:471–475, 1968.
10. Siegel M, Fuerst HT: Low birthweight and maternal viral diseases. A prospective study of rubella, measles, mumps, chickenpox, and hepatitis. JAMA 197:88–92, 1966.
11. Steinberg SP, Gershon AA: Measurement of antibodies to varicella-zoster virus using a latex agglutination test. J Clin Microbiol 29:1527–1529, 1991.
12. Demmler G, Steinberg S, Blum G, Gershon A: Rapid ELISA for detecting antibody to varicella-zoster virus. J Infect Dis 157:211–212, 1988.
13. Larussa P, Steinberg S, Waithe E, Hanna B, Holzmen R: Comparison of five assays for antibody to varicella zoster virus and the fluorescent antibody to membrane antigen test. J Clin Microbiol 25:2059–2062, 1987.
14. Hope-Simpon L: Infectiousness of communicable diseases in the household. Lancet 2:549–551, 1952.
15. Yorke JA, London WP: Recurrent outbreaks of measles, chickenpox, and mumps. Am J Epidemiol 98:469–478, 1973.
16. Krugman S, Goodrich GH, Ward R: Primary varicella pneumonia. N Engl J Med 257:843–845, 1957.
17. Mermelstein RH, Freireich AW: Varicella pneumonia. Ann Intern Med 55:456–463, 1961.
18. Treibwasses JH, Harris RE, Bryant RE, Rhoades ER: Varicella pneumonia in adults: Report of 7 cases and a review of the literature. Medicine 46:409–423, 1967.
19. Weber DM, Pellecchia JA: Varicella pneumonia: Study of prevalence in adult men. JAMA 192:572–573, 1965.
20. Gershon AA, Steinberg S, Brunell PA: Zoster immune globulin: A further assessment. N Engl J Med 290:243–245, 1974.
21. Brunell PA, Ross A, Miller LH, Kuo B: Prevention of varicella by zoster immune globulin. N Engl J Med 22:1191–1194, 1969.
22. Judelsohn RG, Meyers JD, Ellis RJ, Thomas EK: Efficacy of zoster immune globulin. Pediatrics 53:476–480, 1974.
